# Practical considerations for sensitivity analysis after multiple imputation applied to epidemiological studies with incomplete data

**DOI:** 10.1186/1471-2288-12-73

**Published:** 2012-06-08

**Authors:** Vanina Héraud-Bousquet, Christine Larsen, James Carpenter, Jean-Claude Desenclos, Yann Le Strat

**Affiliations:** 1 Département des maladies infectieuses, Institut de Veille Sanitaire, 12 rue du Val d’Osne, 94415 St Maurice, France; 2 Département des Maladies Infectieuses, Institut de Veille Sanitaire, St Maurice, France; 3 Medical Statistics Unit, London School of Hygiene and Tropical Medicine, London, France; 4 Direction Scientifique, Institut de Veille Sanitaire, St Maurice, France

## Abstract

**Background:**

Multiple Imputation as usually implemented assumes that data are Missing At Random (MAR), meaning that the underlying missing data mechanism, given the observed data, is independent of the unobserved data. To explore the sensitivity of the inferences to departures from the MAR assumption, we applied the method proposed by Carpenter *et al.* (2007).

This approach aims to approximate inferences under a Missing Not At random (MNAR) mechanism by reweighting estimates obtained after multiple imputation where the weights depend on the assumed degree of departure from the MAR assumption.

**Methods:**

The method is illustrated with epidemiological data from a surveillance system of hepatitis C virus (HCV) infection in France during the 2001–2007 period. The subpopulation studied included 4343 HCV infected patients who reported drug use. Risk factors for severe liver disease were assessed. After performing complete-case and multiple imputation analyses, we applied the sensitivity analysis to 3 risk factors of severe liver disease: past excessive alcohol consumption, HIV co-infection and infection with HCV genotype 3.

**Results:**

In these data, the association between severe liver disease and HIV was underestimated, if given the observed data the chance of observing HIV status is high when this is positive. Inference for two other risk factors were robust to plausible local departures from the MAR assumption.

**Conclusions:**

We have demonstrated the practical utility of, and advocate, a pragmatic widely applicable approach to exploring plausible departures from the MAR assumption post multiple imputation. We have developed guidelines for applying this approach to epidemiological studies.

## Background

Missing data are ubiquitous in epidemiological and clinical research, and in consequence there is increasing interest in appropriate statistical methods, principally multiple imputation (MI) [1,2]. Multiple imputation techniques available in standard statistical software [3,4] enable parameter estimation under the assumption that missing data are missing at random (MAR), meaning that the missingness mechanism depends on observed data only, and given these no longer on the missing data [5].

Incomplete datasets are usually addressed by a complete-case (CC) analysis restricted to individuals that have no missing data in any of the variables required for the analysis. For etiologic analyses, a complete-case approach leads to a loss in power, but gives valid results if the probability of being a complete-case is independent of the outcome, given the covariates in the model [5,6]. However, if the missingness mechanism depends on the outcome, given the covariates, a complete-case analysis can be biased, even under the MAR assumption [7,8]. Conversely, MI allows individuals with incomplete data to be included in the analysis. It yields valid and efficient inferences under the MAR assumption, even if the missingness mechanism is related to the outcome, provided the imputation model is appropriate [5].

Missing data may also be due to a Missing Not At Random (MNAR) mechanism, also termed non-ignorable, meaning that, given the observed data (including the outcome), the missingness mechanism depends on unobserved data. In practice, it is impossible to distinguish between MAR and MNAR data [9]. When performing multiple imputation under MAR, the estimate of the regression coefficient of a covariate with missing values can be subject to bias when the missingness mechanism of the covariate is MNAR, whether this MNAR mechanism depends on the outcome variable or not [6,7]. The extend of this bias is often greater the stronger the dependence of the missingness mechanism on the outcome [10]. Sensitivity analysis is useful in such cases.

Specifically, where the missingness mechanism for one or more of the covariates depends on the response in the model of interest, a MI analysis assuming MAR is preferable to a CC analysis, especially if additional variables, not in the model of interest, can be included in the imputation model to increase the plausibility of the MAR assumption [11-14]. Nevertheless, the missingness mechanism may additionally depend on unseen values of a covariate, and the estimates of the coefficient of this covariate may be sensitive to this. Knowledge of the direction and extent of this sensitivity is important when drawing conclusions from an analysis. The method we present here allows such sensitivity analysis to be performed rapidly after MI under MAR.

In the statistical literature, both selection models [15] and pattern-mixture models [16] have been proposed for the analysis of data under MNAR assumptions [17,18]. Here, our focus is on selection models, which describe assumptions about the mechanisms causing the missing data and then work through the consequences for inference from the model of interest. Unfortunately, methods for such sensitivity analysis are not implemented in standard statistical software and in their full generality are computationally complex. Thus they are little used in practice [1].

However, a computationally much more straightforward approach to local sensitivity analysis, following MI under MAR, has been proposed by Carpenter *et al.*[19,20]. This ‘selection-based’ approach explores the robustness of inference under local departures from the MAR assumption, meaning that the sensitivity to departure from the MAR assumption can be calculated from the observed data without estimating a full non-ignorable model [21]. Parameter estimates obtained from the imputed datasets assuming MAR are reweighted to represent the distribution of imputations under a MNAR mechanism. Consequently, inferences obtained under the MAR and MNAR assumptions can be compared to assess the robustness of inferences to local departures from the MAR hypothesis.

This method is attractive as it is easy to implement after performing MI, and it has not been reported for observational data to our knowledge. We have therefore applied this method to surveillance data for hepatitis C viral infection collected in France [22]. As a result of this, we further propose guidelines on the use of the method for observational data.

## Methods

The hepatitis C virus (HCV) surveillance system is based on 26 participating hepatology reference centers out of the 31 located in university hospitals throughout France [23]. Since 2000, it has enrolled patients at first referral with HCV chronic infection to monitor changes in characteristics of HCV infection. Here, ‘first referral’ is defined as a patient’s first consultation at the clinic with no prior histologic evaluation of their liver disease [22]. A standardized questionnaire is used to collect epidemiological (date of first referral and last HCV negative test, circumstance of HCV antibody testing, and risk factors), clinical, biological (HCV RNA serum status, HIV and HBV co-infection), and history of excessive alcohol consumption data.

For this study we considered the 4,343 cases that reported having injected or snorted drugs at least once in their whole life. We investigated risk factors predictive of severe liver disease (SLD) at first referral by multivariate logistic regression. SLD was defined as cirrhosis or hepatocellular carcinoma at first referral, as assessed by biochemical tests and morphological evaluation [24]. Note that the risk factor data were collected independently of the outcome of interest.

### Preliminary analyses

Details of the study design and the initial analyses have already been described [22,23]. Six out of the 9 variables retained for the multivariate analysis were incomplete, with a range of missing values from 10 to 26% (Table 1). In the CC analysis, multivariate logistic analysis was reduced to 1,858 individuals (43% of total cases) having no missing data in any of the 9 variables of the analysis. Consequently, we estimated missing values through multiple imputation by chained equations using Stata's user written program *ice*[4] (STATA ® 9.2, Stata Corporation, College Station, Texas, USA). This computationally convenient method is being increasingly used in epidemiology, and does not require any direct assumption on the joint distribution of the variables [25,26]. The imputation algorithm is based on a set of univariate imputation models which, in turn, regress one variable on all the other covariates and the outcome [27].

**Table 1:** Multivariate logistic regression of factors associated with severe liver disease

				**Multivariate analysis**	
Factors	Patients (n = 4 343)	% SLD	% missing data	Complete Case (n = 2 130) aOR* (95% CI*)	Multiple Imputation (n = 4 343) aOR* (95% CI*) *M* = 30 imputed datasets
Period of inclusion					
2001-2003	2330	7.0			
2004-2007	2013	9.5			
Sex					
Female	993	4.2		1.0	1.0
Male	3350	9.3		1.8 [1.1,3.0]	2.0 [1.4,2.9]
Age					
≤ 40 years	2435	3.9		1.0	1.0
> 40 years	1908	13.6		2.2 [1.5,3.3]	2.3 [1.7,3.1]
Time between 1^st^ HCV + test and referral					
< 1 year	1728	6.7			
≥ 1 year	2163	8.7			
Missing	452	11.5	10.4		
Duration of HCV infection at referral^†^					
< 18 years	1709	3.0		1.0	1.0
≥ 18 years	2002	12.5		3.1 [2.0,5.1]	2.6 [1.8,3.7]
Missing	632	8.2	14.6		
History of excessive alcohol intake^‡^					
No	2015	4.5		1.0	1.0
Yes	1847	13.2		2.6 [1.8,3.7]	2.8 [2.2,3.7]
Missing	481	4.4	11.1		
HbsAg status					
Negative	3570	8.3		1.0	
Positive	89	13.5		2.4 [1.0,5.9]	
Missing	684	6.7	15.7		
HIV serostatus					
Negative	3342	8.2			1.0
Positive	294	14.0			1.8 [1.2,2.6]
Missing	707	5.7	16.3		
HCV genotype 3					
No	2083	7.2		1.0	1.0
Yes	1117	10.3		1.5 [1.1,2.0]	1.6 [1.3,2.1]
Missing	1143	7.8	26.3		

*aOR, adjusted Odds Ratio; *CI*, confidence interval; *SLD*, severe liver disease (cirrhosis, hepatocellular carcinoma); *HCV*, hepatitis C virus; *HBsAg*, hepatitis B surface antigen; *HIV*, human immunodeficiency virus.

† Time from suspected year of infection to year of referral to the reference centre. Suspected year of HCV infection is defined as year of the last HCV negative test performed during the drug-use period or year of first drug injection.

‡ >210 g/week for women and >280 g/week for men.

Complete case and multiple imputation analyses were applied to a population of HCV-RNA positive drug users newly referred in hepatology reference centres in France, 2001–2007.

The variables in the imputation model were limited to the 9 variables retained after the univariate analysis. No additional (auxiliary) variables were included because they had either too many missing values or were insufficiently related to the missingness mechanism. A total of 30 imputed datasets were generated. The initial study exploring the risk factors of SLD was performed using a joint analysis of these 30 imputed datasets [22]. Further imputations were performed subsequently for the sensitivity analysis.

### Sensitivity analysis method

Consider a variable (covariate or response) *Y* with missing values. We denote by *Y*_*i*_ the value of *Y* for the individual *i*. Let *R*_*i*_ be an observation indicator variable equal to 1 if *Y*_*i*_ is observed and 0 if otherwise. We assume a logistic model relating the probability of observing *Y* to the underlying (but potentially unseen) value of *Y* itself, adjusted for a vector *X* of covariates:

(1) $$\log\text{it}\text{Pr}(R_{i}=1)=\alpha +\beta X_{i}+\delta Y_{i}\text{.}$$

Under this parametric form assumption, if *δ* = 0, given the fully observed data, the mechanism causing the missing data of *Y* does not depend on *Y*, so that the missing data are MAR. On the contrary, if *δ* ≠ 0, the missingness mechanism depends on the potentially missing *Y*, even taking into account the information in the observed data. Thus the data are MNAR.

In practice, the above logistic regression cannot be performed since, by definition, we do not observe *Y*_*i*_ when *R*_*i*_=0. This implies that a value for *δ* must be chosen, and its effect on inferences from the model of interest explored. With the method we investigate, this can be done using weights which are a simple function of *δ* and the imputed data. We next give an intuitive explanation of the approach.

Suppose M datasets are created by a MI method assuming MAR. For each dataset, we denote by ${\hat{\theta }}_{m}$ the estimate of the parameter of interest (e.g. a regression coefficient). Multiple imputation assuming MAR results in several point estimates which, under Rubin’s rules, are simply averaged for final inference. Thus, the usual MI estimate of *θ* is expressed by:

${\hat{\theta }}_{\text{MAR}}=\frac{1}{M}\sum_{m=1}^{M}{\hat{\theta }}_{m}\text{.}$ (see Appendix for its estimated variance).

Carpenter’s approach works by replacing this simple average by a weighted average, where estimates arising from imputations that are more likely under MNAR are upweighted relative to the others. Under the logistic model for the missingness mechanism described in (1), Carpenter *et al.* show the weights take a particularly simple form [20].

The model (1) hypothesises that, after adjusting for other observed variables, the chance of observing Y per unit change in Y has log-odds ratio *δ*. Then the weight, noted ${\tilde{w}}_{m}\left(\delta \right)$for imputation *m*, (*m = 1,…,M*)*,* is equal to $\exp\left[-\delta \sum_{i\in I_{Y}}Y_{i}^{m}\right]\text{,}$, where *Y*_*i*_^*m*^ (*i* ∈ *I*_*Y*_) is the imputed value of Y for the individual *i* in the dataset *m*, and *I*_*Y*_ is the set of individuals with Y unobserved. The exponential form of the weights comes from the logistic link in equation (1).

Normalized weights calculated for each imputed dataset are expressed by $w_{m}\left(\delta \right)=\frac{{\tilde{w}}_{m}\left(\delta \right)}{\sum_{k=1}^{M}{\tilde{w}}_{k}\left(\delta \right)}$.

The MNAR estimate of *θ* is defined by ${\hat{\theta }}_{\text{MNAR}}\left(\delta \right)=\sum_{m=1}^{M}w_{m}\left(\delta \right)\cdot {\hat{\theta }}_{m}$ (see Appendix for its estimated variance).

Note that if data are MAR, then *δ* = 0, and all imputations are equally weighted as in Rubin’s original rules.

To gain an intuition for these weights, if δ is positive the chance of observing *Y* is greater for more positive *Y*. Thus in the data after imputation under MAR, imputations with small Y will be under-represented. The weights correct this by up-weighting (relative to the other imputed data sets) estimates from imputed data sets where the sum of the imputed values of *Y* is small.

Below, we present MAR estimates for the HCV dataset and explore their robustness to MNAR as *δ* moves away from zero. We further propose practical guidelines for selecting a *δ* value where possible (or at least a plausible range of values for *δ*).

### Framework for sensitivity analysis

Among variables retained in the multivariate analysis (Table 1), we focused on the missingness mechanisms of 3 binary variables. We now discuss epidemiological hypotheses about these mechanisms for each variable in turn.

#### Alcohol consumption

Reporting alcohol consumption may be prone to a social desirability effect, even when past consumption is accounted for. We hypothesized that former heavy drinkers were less likely to report their past alcohol consumption.

#### HIV infection

HIV serostatus could be assessed either by a previous HIV test, where available, or by a test at first referral. Since the prevalence of HCV-HIV co-infection in hepatology reference centers is ~8% and HIV testing is quite systematic, the physician may consider that patients are mainly HCV mono-infected when no positive HIV test is available. We hypothesized that HIV testing is less often reported when patients are HIV negative.

#### HCV genotype 3

HCV genotypes are tested by the referral laboratories of the participating centers. Genotyping might depend on the physicians’ attitudes, but probably not on the unobserved values of the genotype. We nevertheless explored the MNAR assumption.

Consequently, we focused the sensitivity analysis on these 3 variables to assess the robustness of estimates obtained after MI using Carpenter’s method [20]. The sensitivity analysis was applied to each variable separately in turn.

### Practical considerations for sensitivity analysis

Although it is recommended to impute at least 50 datasets [20], we chose to impute 1000 datasets using the Stata *ice* program to illustrate the features of the method. This is double the number used by Carpenter *et al.*[19] in a simulation study to test the method; although imputations are computationally cheap, beyond 1000 the gain, in terms of increased range of the imputation estimates ${\hat{\theta }}_{m}$, is small.

One way to select a value for *δ* is to formally elicit plausible values from experts [28]. An alternative is to explore a range of values consistent with hypotheses concerning the missing data mechanism, such as those outlined above.

We propose the following 4-step approach for choosing an appropriate value for *δ*, and illustrate this using the HCV genotype 3 variable, before applying it to the other variables. Our focus is sensitivity analysis for the parameter of interest i.e. the coefficient of genotype 3 in the post MI multivariate logistic regression explaining SLD (Table 1, rightmost column). Using previous notation, ${\hat{\theta }}_{m}$ is the MAR estimated logistic regression coefficient of genotype 3 in the imputed dataset *m = 1,…,M*.

### Procedure for choosing delta

#### Step 1: Logistic regression to explore the missingness mechanism

Generate an indicator variable for the covariate in question being missing, and use logistic regression to assess association with the outcome and other covariates.

Illustrating with genotype 3, we generate a missing indicator equal to 1 if genotype 3 is observed and 0 otherwise. Using imputed values, we then fit a multivariate logistic regression model to explain the genotype 3 missing indicator; in this model we include the outcome (SLD) and all the covariates included in the initial analysis model, genotype 3 excepted.

The results, shown in Table 2, suggest the missingness mechanism for genotype 3 depends on age and disease duration, but given these is independent of SLD, the outcome in the analysis model.

**Table 2:** Multivariate regression to explain the missing indicator of genotype 3 using covariates

**Genotype 3 missing indicator**	**Regression coefficients**	SE*	P*
Severe liver disease ^†^	−0.05	0.13	0.72
Age	0.16	0.09	0.06
Sex	0.04	0.08	0.63
Disease duration ^‡^	0.19	0.08	0.04
Delay of referral ^4^	0.05	0.08	0.53
Alcohol consumption ^5^	−0.005	0.07	0.94
HIV serostatus	0.02	0.14	0.90
HbsAg status	−0.14	0.23	0.52

* *P*, pvalue; SE, standard error.

† Cirrhosis or hepatocellular carcinoma.

‡ Time from suspected year of infection to year of referral to the reference centre. Suspected year of HCV infection is defined as year of the last HCV negative test performed during the drug-use period or year of first drug injection.

^4^ Time between testing and first referral.

^5^ >210 g/week for women and >280 g/week for men.

#### Step 2: Graphical determination of a delta value

The theoretical justification of the method rests on importance sampling [29]. When using importance sampling, it is not recommended to put all the weight on one, or very few values. The implication is that we should restrict the range of *δ*. Consistent with this, we recommend the following criteria: values of *δ* should be such that the maximum normalized weight is around 0.5, and at least 5 normalized weights are above 1/M (the weight when *δ* = 0) [20]. Thus our MNAR estimate will draw on information from at least 5 imputations, the minimum typically advised in practice. It also reflects practically relevant, yet appropriately local, departures from MAR.

**Figure 1:**
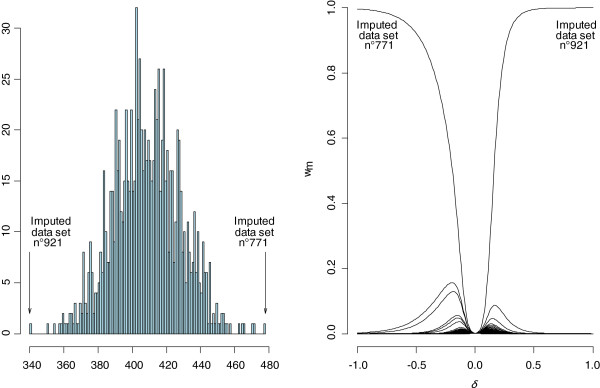
**Graphical determination of a delta value for the variable genotype 3.** Left panel: histogram of the sum of genotype 3 imputed values for each data set and for M = 1000 bases ; extreme values of this sum are 340 in imputed dataset n°921 and 480 in imputed dataset n°771. Right panel: normalized weights (w_m_) for each imputed dataset according to δ.

In practice, we recommend presenting this information in a graph such as Figure 1. The left panel shows a histogram of the sum of the imputed values for genotype 3. Extreme values are 340 in imputed dataset n°921 and 480 in dataset n°771. The right panel indicates normalized weights for each of the M = 1000 datasets by *δ*value. The maximum normalized weight corresponds to the dataset(s) in which the sum of imputed values of *Y* is minimal (dataset n°921) when *δ*>0 or maximal (dataset n°771) when *δ*<0. When *δ*=0, the normalized weight is equal to 1/M because all the ${\tilde{w}}_{m}\left(0\right)$ are equal to 1.

**Figure 2:**
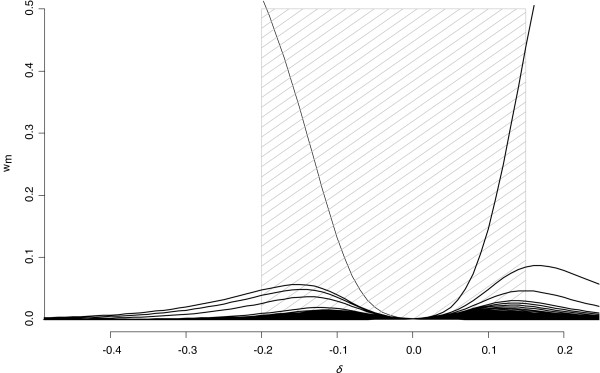
**Normalized weights (w**_**m**_**) for each imputed dataset according to δ for the variable genotype 3.** The hatched zone delineates values of *δ*corresponding to maximum normalized weights equal to 0.5.

Figure 2 shows the central part of the right panel of Figure 1. Following our recommendation above, we retain positive or negative *δ* values that correspond to a maximum normalized weight of ~0.5. This gives a range of [−0.2 to 0.15]. Even at the end of this range, more than 5 normalized weights are > 0.001. The central part of the hatched zone (defined subjectively, although an objective criteria could be set down *a-priori* if desired) corresponds to departures from MAR for which the weights are still approximately equal, so that MAR and MNAR inferences are essentially the same.

#### Step 3: Choice of sign of delta

Here, we choose *δ* to be either the upper or lower end of the range identified in step 2.

For HCV genotype, equation (1) shows the relation between the sign of *δ* and the assumed missingness mechanism: for positive *δ*, the adjusted odds for observing genotype increases if a person’s HCV is of genotype 3; for negative *δ* the converse. In this instance, consistent with the results from step 1 (Table 2), we selected *δ* = 0.15. This means that the adjusted odds of missing data for genotype 3 is 1.2 (exp(0.15)) times greater for individuals infected by a genotype 3 strain than for those infected by other genotypes. For this variable, experience does not strongly suggest a positive or negative *δ*, and results for both are presented below.

#### Step 4: Graphical diagnostic

The re-weighting method is for local sensitivity analysis, because the underlying theory requires the probability distribution of the estimator of the parameter of interest under MAR and MNAR to share the same support (albeit they have different means). This will not generally hold for non-local sensitivity analysis. The ‘range’ of such local sensitivity analyses will depend on the between imputation variance of the estimator, which is indirectly related to the proportion of missing observations. To assess whether, at the chosen value of *δ*, this holds we propose (i) a plot of normalized weight *w*_*m*_ against ${\hat{\theta }}_{m}$, *m = 1,…,M* and (ii) a plot of the estimate under MNAR as the number of imputations increases. In both plots, if the method is to give reliable results, the MNAR estimator should be supported within the distribution of ${\hat{\theta }}_{m}$ obtained by MI under MAR. If all the weight is accruing to estimates at the end of the range of ${\hat{\theta }}_{m}$, this is consistent with the MNAR estimator having a distribution lying outside the range of MAR estimates, i.e. a ‘non-local’ departure from MAR. Under such a non-local MNAR mechanism the estimate of *θ* is most likely beyond the smallest (largest) of the MAR imputation estimates.

**Figure 3:**
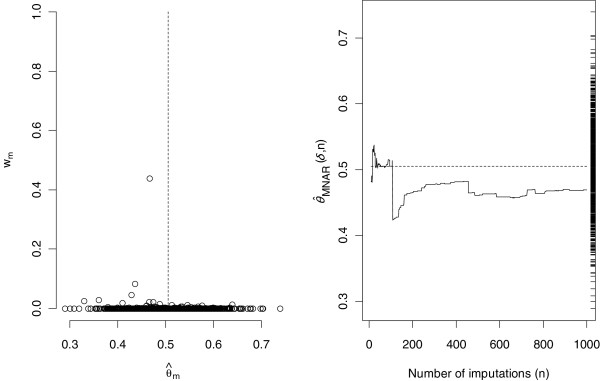
**Analysis of the variable genotype 3 with δ = 0.15.** Left panel: normalized weights (w_m_) versus ${\hat{\theta }}_{m}$(estimated logistic regression coefficient of genotype 3 in the imputed dataset *m*), for each imputed data set. The dash line represents ${\hat{\theta }}_{\mathrm{MAR}}$ (mean of ${\hat{\theta }}_{m}$ over the 1000 imputed datasets). Right panel: running estimate, calculated as the moving average of the ${\hat{\theta }}_{\mathrm{MNAR}}$ according to the number of imputed datasets. On the right axis is plotted the ‘rug’ of the 1000 estimates ${\hat{\theta }}_{m}$ for each imputed dataset. The dash line represents ${\hat{\theta }}_{\mathrm{MAR}}$ (mean of ${\hat{\theta }}_{m}$ over the 1000 imputed datasets).

For the HCV genotype variable the results are shown in Figure 3. The left-hand panel plots the normalized weights versus ${\hat{\theta }}_{m}$ for each imputed data, using *δ*=0.15 (recall ${\hat{\theta }}_{m}$is the regression coefficient estimate obtained under the MAR assumption for each imputed dataset). The right panel plots the MNAR estimate calculated using *n* imputations against the number of imputed datasets noted *n* (*n = 10,…,M*) and defined by:

$${\hat{\theta }}_{\mathrm{MNAR}}\left(\delta ,n\right)=\frac{\sum_{m=1}^{n}w_{m}\left(\delta \right)\cdot {\hat{\theta }}_{m}}{\sum_{m=1}^{n}w_{m}\left(\delta \right)}\text{.}$$

In this case we see that (i) the MNAR estimate appears to settle down as the number of imputations increases and (ii) the MNAR distribution of ${\hat{\theta }}_{}$seems comfortably supported within the MAR distribution (indicated by the ‘rug’ on the right side of the plot).

## Results

The complete-case and MI (assuming MAR) analysis are shown in Table 3. Here we also give the results of sensitivity analysis for the following three variables: HCV genotype 3, HIV serostatus and history of excessive alcohol consumption.

**Table 3:** **Multivariate analysis for the complete case, multiple imputation and sensitivity analysis, with *M*** **= 1000 imputed data sets**

		**Complete Case (CC)**			**Multiple Imputation (MI)**				**Sensitivity Analysis (SA)**				
	Missing values %	aOR 95% CI	SE	CV (%)	aOR 95% CI	SE	CV (%)	VR_MI_(MI *vs* CC)(%)	δ	aOR^*^95% CI	SE	CV (%)	VR_SA_(SA *vs* MI)(%)
Alcohol consumption	11.1	2.32	0.39	17	2.82	0.37	13	21.86	-0.40	2.86	0.37	13	1.29
		[1.66,3.23]			[2.18,3.66]					[2.21,3.70]			
Genotype3	26.3	1.51	0.24	16	1.66	0.23	14	9.70	0.15	1.60	0.21	13	3.56
		[1.10,2.07]			[1.27,2.16]					[1.23,2.06]			
HIV	16.3	1.56	0.41	27	1.80	0.34	19	15.52	0.70	1.91	0.36	19	6.12
serostatus		[0.92,2.62]			[1.24,2.61]					[1.32,2.76]			

Note. aOR, odds ratio adjusted on sex, age, duration of HCV infection, alcohol consumption and HIV serostatus; aOR^*^, odds ratio obtained from the MI adjusted odds ratio estimates.

*CI*, confidence interval; *CV*, coefficient of variation of the aOR; *VR*_*MI*_ , variation rate of the aOR for CC and MI analyses; *VR*_*SA*_ , variation rate of the aOR for MI and senstitivity analyses.

Covariates included in the model were: sex, age, duration of HCV infection, alcohol consumption, genotype 3 and HIV serostatus. Sensitivity analysis: the weighting process was applied to alcohol consumption, genotype 3 and HIV serostatus indepedently.

For HCV genotype 3, we derived the value of the sensitivity parameter *δ* above, to illustrate our four step approach. We applied the same approach to HIV serostatus and alcohol consumption.

**Figure 4:**
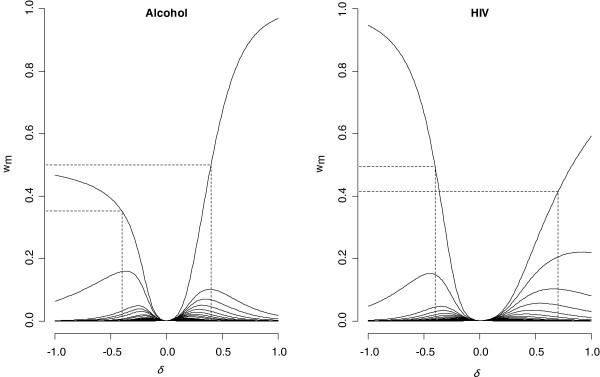
**Normalized weights (w**_**m**_**) for each imputed dataset according to δ for the variables alcohol consumption and HIV serostatus.** Left panel: the interval for *δ* is restrained to [−0.4;0.4]. Right panel: the interval for *δ* is [−0.4;0.7].

For alcohol consumption, step 1 showed the probability of observing this depends on the outcome (SLD status). Step 2 identified the range for *δ* of [−0.4;0.4] (left panel of Figure 4). Consistent with step 1 and experience, the odds of observing alcohol intake is higher if it is not excessive, we chose *δ*= −0.4. The interpretation is that, after adjustment for other variables, the odds of observing alcohol history is reduced among those with a history of excessive intake by 0.7 = exp(−0.4).

For HIV serostatus, step 1 showed the probability of observing this depends on the outcome (SLD status). Step 2 identified a range of [−0.4;0.7] (right panel of Figure 4). Taking the results from step 1, and given that in similar contexts the chance of observing HIV infection is higher for HIV positive individuals, we chose *δ* = 0.7. The interpretation is that, after adjustment for other variables, the odds of observing HIV serostatus is 2.0 = exp(0.7) times higher if HIV serostatus is positive.

For these three variables, the diagnostic in step 4 was acceptable. Adjusted odds ratios (OR) are shown in Table 3. Note the same multivariate model including sex, age, duration of HCV infection, alcohol consumption, genotype 3, and HIV serostatus, was applied for each analysis. The sensitivity analysis was applied to each of the 3 variables in turn.

Two criteria are useful to interpret the adjusted odds ratios in the 3 analyses:

1 The coefficient of variation (CV) of the OR gives its normalized measure of dispersion. For the 3 variables, it is clearly reduced after MI and remains stable after reweighting.

**Figure 5:**
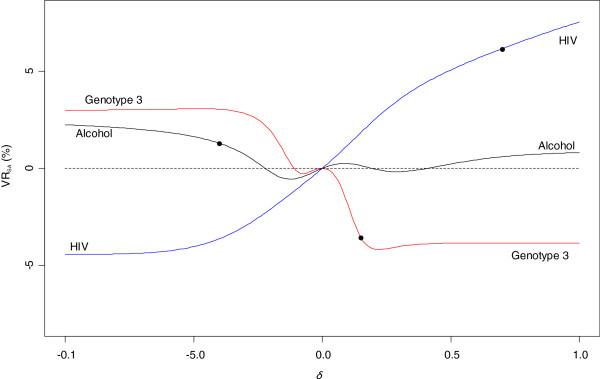
**Variation rate according to δ after sensitivity analysis (VR**_**SA**_**) for genotype 3, alcohol consumption and HIV serostatus**. The black points correspond to the VR_SA_ calculated for the value of δ retained for each variable (genotype 3 δ = 0.15, alcohol δ = −0.4 and HIV δ = 0.7).

2 The variation rate (VR) assesses the relative change between the $O{\hat{R}}_{\mathrm{MNAR}}$ and the $O{\hat{R}}_{\mathrm{MAR}}\text{,}$ and is defined by $VR_{\mathrm{SA}}=100\times (O{\hat{R}}_{\mathrm{MNAR}}-O{\hat{R}}_{\mathrm{MAR}})/O{\hat{R}}_{\mathrm{MAR}}$. Similarly, we define a variation rate named VRMI that displays the relative variation of the OR obtained after CC and MI analyses. VRMI varies from 9.7% for genotype 3 to 15.5% for HIV and 22% for alcohol. VRSA is given for the value of δ selected for each variable. Its value is relatively small for alcohol (1.3%) and genotype 3 (3.5%) but larger for HIV at 6.6%. The VRSA is relatively stable as δ varies in [−1;1] for alcohol and genotype 3, but continues to increase for HIV (Figure 5).

## Discussion

With missing data, all analyses and corresponding inferences rest on inherently untestable assumptions about the missingness mechanism. Therefore, sensitivity analyses, where we explore the robustness of inferences as assumptions change, are important.

The method presented here enables rapid local sensitivity analysis to inferences obtained via MI under MAR. It works by upweighting imputations which are more plausible under MNAR; under a logistic model for the missingness mechanism, these weights take a particularly simple form.

While the sensitivity analysis is local, it nevertheless provides important information on the duration and impact of departures from MAR on inference, while avoiding the computational complexity of full joint modeling. Its accuracy for local sensitivity analysis has been confirmed elsewhere [20,30].

Here, we have developed and illustrated the practical utility of the approach, proposing a 4-step process for choosing a value for the sensitivity parameter. We now discuss the results. Note that all three variables are binary, so the scale for delta is the same.

For genotype 3, step 1 of our process shows that among individual with complete records on variables apart from genotype 3, the probability of observing this variable does not appear to be related to the outcome in the model of interest (severe liver disease) (Table 2). The sensitivity analysis allows this probability additionally to depend on the underlying value of genotype 3 (present or absent). Table 3 and Figure 5 show inference is insensitive to this, indeed for plausible delta the estimate moves back towards the complete case estimate, consistent with what would be expected if the additional MNAR dependence does not materially change the lack of dependence of the chance of observing genotype 3 on severe liver disease.

Regarding alcohol consumption, our hypothesis was that patients will be less willing to report past excessive alcohol consumption because of the associated social stigma. However, the literature is not unanimous on this [31-34]. Reporting alcohol consumption is strongly related to the sociodemographic characteristics [35] that can be included in the imputation model in order to reduce non-response bias. We only included age and sex in the imputation model because other sociodemographic variables were not related to the missingness mechanism. Our step 1 showed that the probability of observing alcohol does depend on the outcome, after taking into account other covariates. This is consistent with the relatively large change in the alcohol covariate under MAR (Table 3). The sensitivity analysis allows this probability additionally to depend on the alcohol value. Table 3 and Figure 5 show inference is relatively insensitive to this, which is consistent with what would be expected if the additional MNAR dependence does not materially change the dependence of the chance of observing alcohol consumption on severe liver disease.

For HIV co-infection, hepatologists in reference centers tend to consider their patients as being HCV mono-infected because HCV-HIV co-infected patients are usually referred to infectious diseases departments in France. Under the default assumption of mono-infection, there would thus usually be less impetus to test for or record HIV status. Thus we felt it was more likely to be observed if it was present, i.e. serostatus was positive, hence our positive value for *δ*. Step 1 showed that the probability of observing HIV status does depend on outcome, after taking into account other covariates. This is consistent with the relatively large change in the coefficient under MAR. The sensitivity analysis allows this probability additionally to depend on the HIV status. Table 3 and Figure 5 show inference is sensitive to this, suggesting that if the mechanism is MNAR with increased chance of observing HIV status for those who are positive, the association in the model of interest is stronger and more significant than analysis under MAR would suggest.

In summary, for these data collected from a French surveillance system for hepatitis C infection, CC analysis is plausibly biased, as the data suggest dependence of the chance of observing values on the outcome, even given the covariates. Thus analysis under MAR, via MI, is preferable. Our sensitivity analysis shows that for local departures from MAR, inference for the genotype 3 and alcohol consumption is little changed, while the effect of HIV status is underestimated if, given the observed data, the chance of observing HIV status is higher when this is positive.

The approach we have described here can also be applied to explore the situation when there is an interaction between, say, the response (disease status) and the chance of a risk factor being observed. In this case we may have two sensitivity parameters, one for each group (disease status), or possibly a single parameter representing the difference between these. Since some evidence for this has been found [36] this is a natural area for future work.

## Conclusion

This sensitivity analysis provides a fast, albeit approximate, way to assess the robustness of inferences to the MAR assumption, avoiding the need for further imputation and model fitting to the imputed datasets. In this paper we have proposed a 4-step process for using this method in practice. We have demonstrated the application of this method and the interpretation of the results. Faced with non-trivial proportions of missing data, we encourage readers to apply the method in their own analyses.

## Appendix

Let *M* the number of imputed datasets, ${\hat{\theta }}_{m}$ the estimated parameter of interest in the imputed dataset *m*, *m = 1,…,M*, and ${\hat{\sigma }}_{m}^{2}$ its associated variance estimate. The estimated MAR variance of ${\hat{\theta }}_{\mathrm{MAR}}$ is:

${\hat{V}}_{\mathrm{MAR}}\left({\hat{\theta }}_{\mathrm{MAR}}\right)={\hat{V}}_{W}\left({\hat{\theta }}_{\mathrm{MAR}}\right)+\left(1+\frac{1}{M}\right)\cdot {\hat{V}}_{B}\left({\hat{\theta }}_{\mathrm{MAR}}\right)$, where ${\hat{V}}_{W}\left({\hat{\theta }}_{\mathrm{MAR}}\right)=\frac{1}{M}\sum_{m=1}^{M}{\hat{\sigma }}_{m}^{2}$ and ${\hat{V}}_{B}\left({\hat{\theta }}_{\mathrm{MAR}}\right)=\frac{1}{M-1}\sum_{m=1}^{M}{\left({\hat{\theta }}_{m}-{\hat{\theta }}_{\mathrm{MAR}}\right)}^{2}$.

The estimated MNAR variance of ${\hat{\theta }}_{\mathrm{MNAR}}$ for a chosen *δ*value is: ${\hat{V}}_{\mathrm{MNAR}}\left({\hat{\theta }}_{\mathrm{MNAR}}\left(\delta \right)\right)\approx {\hat{V}}_{W}\left({\hat{\theta }}_{\mathrm{MNAR}}\left(\delta \right)\right)+(1+\frac{1}{M})\cdot {\hat{V}}_{B}\left({\hat{\theta }}_{\mathrm{MNAR}}\left(\delta \right)\right)$, where ${\hat{V}}_{W}\left({\hat{\theta }}_{\mathrm{MNAR}}\left(\delta \right)\right)=\sum_{m=1}^{M}w_{m}\left(\delta \right)\cdot {\hat{\sigma }}_{m}^{2}$ and ${\hat{V}}_{B}\left({\hat{\theta }}_{\mathrm{MNAR}}\left(\delta \right)\right)=\sum_{m=1}^{M}w_{m}\left(\delta \right)\cdot {\left({\hat{\theta }}_{m}-{\hat{\theta }}_{\mathrm{MNAR}}\left(\delta \right)\right)}^{2}$.

## Competing interests

The authors declare that they have no competing interests.

## Authors' contributions

VHB implemented the method, performed analyses and interpretation of data, and drafted the manuscript. CL participated to the data gathering, contributed to interpretation of the study and revised the manuscript. JC contributed to the application of the method, and helped to draft the manuscript. JCD helped conduct the epidemiological discussion, and critically revised the manuscript. YLS directed the implementation of the method, contributed to the statistical supervision, and revised the manuscript. All authors read and approved the final manuscript.

## Pre-publication history

The pre-publication history for this paper can be accessed here:

http://www.biomedcentral.com/1471-2288/12/73/prepub
